# Telehealth use in the well-child health setting. A systematic review of acceptability and effectiveness for families and practitioners

**DOI:** 10.1016/j.ijnsa.2024.100277

**Published:** 2024-12-03

**Authors:** Kim Howland, Kristina Edvardsson, Helen Lees, Leesa Hooker

**Affiliations:** a Judith Lumley Centre, School of Nursing and Midwifery, La Trobe University, Melbourne, Victoria, Australia; b Department of Rural Health Sciences, La Trobe Rural Health School, La Trobe University, Bendigo, Victoria, Australia

**Keywords:** Community Health Nursing, Child Health, Community Health Centres, Maternal-Child Health Centres, Remote Consultation, Rural Health, Telemedicine, Telephone, Videoconferencing

## Abstract

**Background:**

Universal well-child health nursing services in high-resource countries promote the health and well-being of children and families while preventing health inequities. The COVID-19 pandemic and technological advancements have led to the increased use of telehealth in this field. To enhance policies and practices, it's important to understand the acceptability and effectiveness of telehealth, as well as the barriers and enablers to its implementation, and to determine when its use is appropriate and safe.

**Objective:**

To explore the global evidence on the use of telehealth in well-child health settings within high-income countries. Focussing on the effectiveness and acceptability of telehealth, along with the factors affecting its implementation and the considerations for safety from the perspectives of both providers and families.

**Design:**

A mixed methods systematic review

**Methods:**

A systematic review was conducted following PRISMA guidelines. The inclusion criteria for the review included: primary research papers written in English, conducted in high-income countries, focused on telehealth in the well-child health setting, and involving children under six years of age. The search, which was completed in July 2023, systematically explored the CINAHL, ProQuest Central, PubMed, and Web of Science bibliographic databases. Studies were critically appraised for quality, and relevant data extracted. A convergent segregated approach was employed to synthesise both quantitative and qualitative data, which is presented in a narrative format.

**Results:**

A total of 4,354 records were identified and screened, and 169 full-text papers were assessed for eligibility, resulting in 20 papers for inclusion. Telehealth acceptability among families was reported in 13 of the 20 studies reviewed, with participants expressing high satisfaction regarding its use as a complement to standard care. Only three studies examined practitioners' acceptance, revealing mixed responses. Effectiveness was observed in 15 studies, with no significant differences found between the control and telehealth groups, suggesting that telehealth may achieve outcomes like those of standard care. Four studies identified both enablers and barriers to the implementation of telehealth, though none addressed concerns regarding safety and appropriateness.

**Conclusions:**

Telehealth shows promise for well-child health services, but there is limited evidence of its effectiveness and safety. The COVID-19 pandemic increased its use, yet risks need further exploration. To validate telehealth in this field, we must identify effective applications, tackle implementation barriers, and ensure client safety. Additional research is essential for developing evidence-based policies for future practices.

**What is already known**

• Telehealth is an effective model of care in the acute health setting.
• Telehealth research in paediatric settings has been limited to treatments of clinical populations.
• Telehealth in preventative well-child health services research is limited.

Alt-text: Unlabelled box

**What this paper adds**

• Telehealth appears an acceptable alternative model of care in the well-child health setting.
• Further exploration is required on the effectiveness outcome measures and the safety and potential harms of telehealth in the well-child health setting.

Alt-text: Unlabelled box

## 1 Background

### 1.1 Well-child health setting

Child health inequity impacts the health and well-being of generations and wider society (Woolfenden et al., 2018; Marmot, 2016). This understanding has led to the establishment of well-child health services designed to optimise the social, psychological, and physical health of children under school age, and their families. The service provider engages with families who are invited to attend scheduled visits for health promotion and surveillance, to optimise the health and well-being of the child (Braithwaite et al., 2021). The consultations generally include developmental screenings, growth monitoring, parental support, anticipatory guidance and health promotion (Wennergren et al., 2023). Well-child health services are offered across most high-income nations, although the format and providers of these services may differ across settings (Garg et al., 2019). Whilst there is no universally recognised title for these services, they are generally community-based, primary prevention programs and include family-centred care, maternal and child health and well-being assessments, anticipatory guidance, developmental surveillance, psychosocial assessments, and a range of screening and health promotion activities (Braithwaite et al., 2021; Garg et al., 2019).

The frequency of recommended well-child health visits varies across high-income countries, from three to four scheduled appointments in the United Kingdom in comparison to 14–18 appointments offered in Sweden (Wood and Blair, 2014). The qualification of the service provider also varies, however, generally, the service is either nurse-led and focused on child development, well-being, and family functioning or physician-led and focused on physical health (Wood and Blair, 2014). Service delivery also varies from phone support, clinic visits, and increasingly, telehealth.

During the global COVID-19 pandemic, as an infection control strategy, well-child health services were forced to transition from in-person to telehealth consultations. While telephone consultations have always been a component of well-child services, they have not been regarded as a replacement for traditional face-to-face care (Gudipudi et al., 2022).

### 1.2 Telehealth

Telehealth can be defined as the use of telecommunications, such as telephones, video links, and webpages to provide health care (Dorsey and Topol, 2016). Telehealth can be further defined into synchronous and asynchronous service provision. Synchronous telehealth is ‘live’ virtual appointments, for example, consultations via a video link or telephone which entail interaction between provider and consumer. Asynchronous telehealth communication is one-way or non-interactive, such as text messages, applications, and webpages (Hah and Goldin, 2022). Telehealth is often used in rural settings (Harkey et al., 2020) and for acute medical conditions, such as the provision of remote care to a stroke or trauma patient in hospital emergency departments (Dorsey and Topol, 2016). To date, the evidence regarding the use of telehealth with parents and children has largely involved the treatment of clinical paediatric populations (e.g., delivering therapy-based interventions for children with Autism and Prader-Willi syndrome) (Bearss et al., 2018; Zyga et al., 2018) and applied behavioural analytic procedures for parental training and parenting interventions (Tomlinson et al., 2018). Telehealth service delivery in the preventative health area is less explored.

To our knowledge, there has been no synthesis of the literature regarding the acceptability and effectiveness in terms of developmental assessment, health promotion and prevention, of using telehealth technology for well-child health service provision. Previous systematic reviews regarding the use of telehealth technology in paediatric populations have focussed on training individuals to undertake an intervention or program in other healthcare disciplines such as behavioural psychology (Tomlinson et al., 2018) and palliative care (Bradford et al., 2013).

### 1.3 Well-child health services and telehealth

Increasing technological advances have allowed telehealth expansion to well-child health consultations, education, information sharing, and practitioner supervision (Dorsey and Topol, 2016; Tomlinson et al., 2018). Especially since the COVID-19 pandemic, where public health measures, to reduce the spread of disease, required health practitioners to be agile and modify service delivery from traditional face-to-face models to telehealth via digital platforms (Adams et al., 2020; Jack et al., 2021).

These advances in technology create the opportunity for consultations to be provided via telehealth in the universal well-child health setting and allow for parents to access their child's developmental status and to address any concerns. If acceptable, safe, and effective, this could provide a cost-efficient way of delivering these services.

For example, in Australia where some of the most prolonged and strict lockdown measures were experienced (Hui et al., 2022), the COVID-19 global pandemic forced universal well-child health services to modify traditional face-to-face and telephone consultations, to comply with public health orders, and reduce the spread of the disease (Adams et al., 2020). As a result, synchronous telehealth via telephone or video link was offered to families instead of the traditional face-to-face service model (Gudipudi et al., 2022).

The rapid change of service delivery from the traditional to an online model was introduced and in many cases without any workforce preparation, education, or change management process (Gudipudi et al., 2022). In Australia, four years into the pandemic, synchronous telehealth is now actively used for a variety of well-child health service offerings including parent education, group work, and some maternal and child health consultations (Victorian Government, 2022). This is an emerging mode of practice, yet the potential harms of distal assessment have not been explored, nor has telehealth feasibility, acceptability, and effectiveness in this setting.

Given service delivery changes, technological advances, and evidence gaps (Wijesooriya et al., 2020), we conducted a systematic review to explore the evidence base on the use of synchronous telehealth, including video link and telephone consultations, with families with children under the age of six years in the well-child health setting and answer the following sub-questions:

1. Is synchronous telehealth an acceptable model of care for both practitioners and families with children under six years of age in the well-child health setting?
2. Is synchronous telehealth a safe and effective model of care for the well-child health setting, regarding developmental assessment, health promotion and prevention for children under six years and age and their families?

To improve policy and practice, we need a better understanding of the acceptability and effectiveness of telehealth, as well as the barriers and enablers to its implementation, and to know when it is appropriate and safe to use.

## 2 Methods

### 2.1 Design

A systematic review following PRISMA guidelines (Page et al., 2021) was completed to ensure a comprehensive examination and reporting of the review process.

### 2.2 Search method

A search strategy was developed with the support of a research librarian, to systematically explore the CINAHL, PRO QUEST CENTRAL, PUB MED and WEB OF SCIENCE bibliographic databases. Using key terms such as maternal and child health service, maternal and child health nurs*, child and family health service, child and family health nurs*, telehealth, telemedicine, telenurs*, newborn*, infant*, Toddler* (see Supplementary File 1). The search of databases was initially completed in July 2022 and updated in July 2023. Search results were imported into Covidence software (Covidence systematic review software, 2022) for ease of subsequent reviewing. The following screening criteria were applied:

#### 2.2.1 Inclusion

• Children under six years of age (and/or families of children under six years).
• Interventions using synchronous telehealth (defined as virtual appointments/consultations such as video link or telephone).
• Studies from high-income countries (Countries defined as a World Bank high-income economy).
• Well-child health services conducted in the community (any community-based primary and prevention health service for children and their families).
• Primary research papers of any study design in English, with full text available.
• Open date range.

#### 2.2.2 Exclusion

• Unwell children (and/or families of only unwell children).
• Children with diagnosed developmental, behavioural, and socio-emotional problems.
• Children (and/or families of children) over six years of age (and/or in the school setting).
• Interventions using asynchronous telehealth (defined as one-way communication, such as text messages, applications, and webpages).
• Acute medical setting.
• Systematic reviews, secondary research, conference papers, opinion papers, letters to the editor, protocol papers, and telehealth education for healthcare providers.

### 2.3 Search outcome

In total 4354 records were identified and screened once duplicates were removed. Title and abstract screening were completed simultaneously by two authors (KH and KE/LH/HL).

**Fig. 1:**
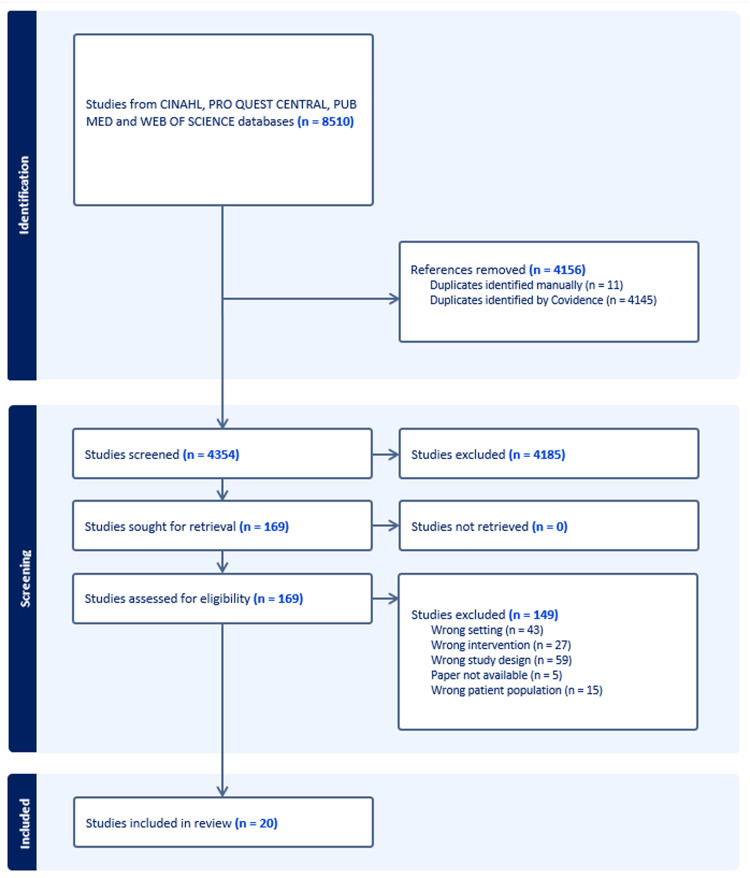
Prisma Flowchart - Utilisation of Synchronous telehealth in the universal well child setting.

Conflicts were resolved by a third reviewer (KE and LH, whichever was the alternative reviewer). On completion of title and abstract screening, 169 full-text papers were reviewed by KH and KE/LH, with conflicts again resolved by the alternative reviewer. This process resulted in 20 papers being included in this systematic review. The screening process is outlined in the PRISMA flow chart Fig. 1).

### 2.4 Quality appraisal

The Joanna Briggs Institute Critical Appraisal Checklists (Aromataris et al., 2024), were used to assess the quality of the 20 studies included in this review. Studies were not excluded based on quality. A score was allocated to each study by KH (see Supplementary File 2). The appraisal scores were reviewed, assessed, and agreed upon by KE and LH. These quality assessments were used to inform review findings. A study was considered high quality if it received a score of ≥10 out of 13 using the randomised controlled trial (RCT) checklist, ≥six out of nine using the quasi-experimental checklist, ≥seven out of 10 using the qualitative checklist, and ≥six out of eight using the cross-sectional studies checklist. Studies were considered medium quality if they received a score between 5 and 9 using the RCT checklist and low quality if they scored ≤5. Quasi-experimental studies were considered medium quality if scored between 3 and 5 and low quality if they scored ≤3 using the quasi-experimental checklist. Using the qualitative checklist medium quality studies scored between 3 and 6 and low-quality studies ≤3. Cross-sectional studies were considered medium quality if they scored between 3 and 5 and low quality if they scored ≤3 using the cross-sectional checklist.

### 2.5 Data extraction

A bespoke data extraction template (Table 1) was developed to identify the nature and scope of telehealth in the well-child setting and assess the relevance of the papers to the review research questions. Table 1 outlines the details of each of the articles under the following headings: citation, country, study aim/s, design, intervention, telehealth mode, sample, setting, healthcare provider and key findings. Data were extracted by KH and reviewed by LH and KE for relevance and accuracy.

**Table 1:** Data Extraction.

Citation	Country	Aim	Design	Intervention	Telehealth Mode	Sample	Setting	Health Care Provider	Key Findings
Agazzi, H., Hayford, H., Thomas, N., Ortiz, C., & Salinas-Miranda, A. (2021). A nonrandomized trial of a behavioral parent training intervention for parents with children with challenging behaviors: In-person versus internet-HOT DOCS. *Clin Child Psychol Psychiatry, 26*(4), 1076–1088. https://doi.org/10.1177/13591045211027559	USA	To describe; the adaptation of the i-HOT DOCS program to telehealth and compare outcomes (child behavior, parenting stress, and program satisfaction) between standard and telehealth delivery.	Quantitative - quasi-randomised controlled trial.	Adaption of a behavioral parent training program from face-to-face to telehealth delivery.	Video-link (MS Teams).	*N*= 366 (eligible parents with children 2–5 years. 317 (86.6 %) completed the evaluation)).	Community setting - In-home telehealth.	Six Certified Instructors including four HOT DOCS professional trainers (an infant–toddler developmental specialist, a pediatric psychologist, a school psychology graduate student and two clinical psychology interns).	Effectiveness- higher mean scores for the in-person program compared to telehealth. -No significant difference across the two program modes in the post-test score differences for child behavior and caregiver stress.
Beaulieu, R., & Humphreys, J. (2008). Evaluation of a telephone advice nurse in a nursing faculty managed pediatric community clinic. *Journal of Pediatric Healthcare, 22*(3), 175–181. https://doi.org/10.1016/j.pedhc.2007.05.006	USA	To examine the effect of nursing telephone advice on parent/caregiver satisfaction with the service and care access.	Quantitative - cross-sectional study (pre-post design).	A nursing telephone service for parents/caregivers.	Telephone.	*N* = 14 parents/caregivers (13 completed pre-implementation of the questionnaire). *N* = 20 parents/caretakers completed the postimplementation questionnaire.	Community primary care pediatric-based clinic in an ethnically diverse and low-income area.	Nurse (specially trained in providing telephone advice).	Acceptability -High satisfaction with the telephone service and nursing advice.
Bock, M. J., Kakavand, K., Careaga, D., & Gozalians, S. (2021). Shifting from in-person to virtual home visiting in Los Angeles County: Impact on programmatic outcomes. *Matern Child Health J, 25*(7), 1025–1030. https://doi.org/10.1007/s10995–021–03169–5	USA	To assess the impact of the shift from in-person home visiting to telehealth due to the COVID-19 pandemic.	Qualitative - a retrospective program review of engagement outcomes before and during the COVID-19 pandemic.	Utilisation of telehealth in a voluntary universal antenatal and postnatal home visiting program.	telephone or via video conferencing	*N* = 12,0000 families annually	In the home (voluntary home visiting across 14 sites).	The program-trained home visitor (nurse) and a parent coach.	Effectiveness - Increased missed visits (stress of home schooling etc.). -Increased number of completed home visits overall (due to saved time not travelling). -Decreased length of time for visits (phone shorter than video). -Assessments and completion not affected.
Ciccia, A. H., Whitford, B., Krumm, M., & McNeal, K. (2011). Improving the access of young urban children to speech, language and hearing screening via telehealth. *J Telemed Telecare, 17*(5), 240–244. https://doi.org/10.1258/jtt.2011.100810	USA	To explore the feasibility, satisfaction and reliability of low-cost videoconferencing for speech, language, and hearing screening for children up to six years of age in urban community health clinics.	Qualitative - Exploratory/feasibility study.	Telehealth provided clinical speech, language, and hearing services at community health clinics from September 2007 to June 2009.	Video link (skype).	*N* = 411 audiology/speech-language pathology video conferencing screenings during the two-year study period. Participants were families with children up to six years of age.	Urban community health clinics.	2 speech-language pathology or audiology students.	Acceptability Screening speech, language and hearing development in young children in urban community health clinics using telehealth is feasible, reliable and supported by the community.
Furukawa, R., Driessnack, M., & Kobori, E. (2020). The effect of video-mediated communication on father-infant bonding and transition to fatherhood during and after Satogaeri Bunben. *Int J Nurs Pract, 26*(4), e12828. https://doi.org/10.1111/ijn.12828	Japan	To explore video-mediated communication during and after Satogaeri Bunben effect on father-infant bonding and transition to fatherhood.	Mixed methods.	Video-mediated communication during the traditional cultural separation of parents, late pregnancy to 1-month post-birth.	Video link (Skype).	*N* = 29 couples (27 fathers completed the study.	traditional separation of couples - mother in her parent's home, father in marital home.	No healthcare provider.	Effectiveness - Increased frequency of father's video-link visitation post childbirth. Acceptability Fathers’ journals (who used Video-link) noted positive comments about the infant's visual cues and moving images, illustrating a sense of “virtual co-presence.”
Gallegos, D., Cromack, C., & Thorpe, K. J. (2018). Can a phone call make a difference? Breastfeeding self-efficacy and nurse responses to mother's calls for help. *Journal of Child Health Care, 22*(3), 433–446. https://doi.org/10.1177/1367493518757066	Australia	To identify characteristics of breastfeeding (BF) support telephone calls for their self-efficacy.	Qualitative - Thematic analyses.	Characteristics of a telephone call that support the caller's breastfeeding self-efficacy.	Telephone.	*N*= 149. Callers accessing the Child Health Line seeking advice on infant feeding. From urban, regional, and remote Australian locations. Predominantly mothers (93 %,*n* = 138), small proportion of fathers (6 %, *n* = 7). other family members/ friends (1 %, *n* = 2).	Telephone line.	Maternal and child health nurses.	Acceptability BF self efficacy affected by medical and moral discourse. Three broad themes reflecting characteristics of support with increased self-efficacy; -Privileging the mother, -Teamwork -Credible Affirmation.
Guiberson, M., Rodríguez, B. L., & Zajacova, A. (2015). Accuracy of Telehealth-Administered Measures to Screen Language in Spanish-Speaking Preschoolers. *Telemed J E Health, 21*(9), 714–720. https://doi.org/10.1089/tmj.2014.0190	USA	To evaluate, hybrid telehealth classification accuracy measures used to screen the language development of Spanish-speaking preschool-age children; to determine screening measures and standardized language assessment scores relationship; to examine if the individual screening measures are accurate; and to use the hybrid telehealth approach to describe the accuracy of the combined screening measures presented.	Quantitative - Cross-sectional study.	Hybrid telehealth (synchronous videoconferencing and videocasting technology) and traditional pen and paper forms.	Video-link.	*N* = 82 children (37 - 69 months of age and their families).	Preschool assessment room.	Spanish speaking speech-language pathologists.	Effectiveness -Classification accuracy of combined measure using hybrid telehealth model. -Preliminary evidence for telehealth models effectiveness in screening language development of Spanish-speaking children.
Gund, A., Sjöqvist, B. A., Wigert, H., Hentz, E., Lindecrantz, K., & Bry, K. (2013). A randomized controlled study about the use of eHealth in the home health care of premature infants. *BMC Medical Informatics and Decision Making, 13*(1), 22–22. https://doi.org/10.1186/1472–6947–13–22	Sweden	To investigate whether video conferencing or a web application improves satisfaction in parents’ taking care of a premature infant at home and decreases the requirement of home visits. In addition, nurses' attitudes toward using these tools.	Quantitative - Randomized Controlled Trial.	12 families had home health care supplemented with the use of a web application and 9 families had home health care supplemented with video conferencing using Skype.	Video link (skype).	*N* = 34 families with premature infants being discharged home.	In-home care (standard care, standard care & web-based support, Standard care & virtual calls).	Nurses – special care nursery.	Acceptability -Families were satisfied with the web app and video conferencing. -Nurses were mostly motivated to use the web application and telehealth but some were reluctant and avoided using it.
Kobayashi, H., & Sado, T. (2019). Satisfaction of a new telephone consultation service for prenatal and postnatal health care. *J Obstet Gynaecol Res, 45*(7), 1376–1381. https://doi.org/10.1111/jog.13987	Japan	To understand if a telephone service provided by a nurse, with access to a clinician, is feasible, acceptable, and satisfactory.	Quantitative - cross-sectional pilot study.	Nursing telephone-based consultation for pre and postnatal care.	Telephone.	*N*= 26 (12 antenatal, 11 postpartum).	Telephone consultation service.	Nurse Practitioner.	Acceptability -Satisfaction with the nursing telephone service (95.7 %). -Positive view of feasibility and acceptability. -respondents concerned with the accuracy, completeness and consistency of information provided by the nursing telephone service.
Lindberg, I., Christensson, K., & Ohrling, K. (2009). Parents' experiences of using videoconferencing as a support in early discharge after childbirth. *Midwifery, 25*(4), 357–365. https://doi.org/10.1016/j.midw.2007.06.002	Sweden	To describe parents’ postnatal experiences when discharged early using videoconferencing.	Mixed Methods.	Videoconferencing for follow-up contact with a midwife in comparison to telephone.	Video-link.	*N*= 11 couples (All 11 couples answered the questionnaires; 2 did not complete interviews; 9 couples/new parents participated in the interviews).	Maternity department and new parents in their homes.	Midwife.	Acceptability In cases of early discharge after childbirth, videoconferencing can facilitate a meeting with the midwife to guide new parents in their transition into parenthood.
Morony, S., Weir, K., Duncan, G., Biggs, J., Nutbeam, D., & McCaffery, K. (2017). Experiences of Teach-Back in a Telephone Health Service. *Health Literacy Research and Practice, 1*(4), 173–181. https://doi.org/http://dx.doi.org/10.3928/24748307–20170724–01	Australia	To explore the experiences of tele-nurses at a maternal and child health helpline using Teach-Back and the caller experiences.	Qualitative - semi-structured guided focus groups and telephone interviews. NB this was part of a larger RCT involving 637 callers to the helpline.	Phone consultations using the 'teach back' method.	Telephone.	*N*= 15 MCH nurses were trained to use Teach-Back (13 nurses participated) and 8 callers.	Telephone helpline.	Maternal and child health nurses.	Acceptability -Nurses found the Teach-Back method was helpful to invite questions from consumers, summarize information, review action plans, close calls -some nurses also found it was helpful to empower and calm (anxious) callers. -Effect on call duration was mixed. -Consumer reports of Teach-Back were limited (*n*= 8) but mostly positive.
O'Connor, K. O. S., Mowat, D. L., Scott, H. M., Carr, P. A., Dorland, J. L., Tai, K. F. W., Steel O'Connor, K. O., Mowat, D. L., Scott, H. M., Carr, P. A., Dorland, J. L., & Young Tai, K. F. W. (2003). A randomized trial of two public health nurse follow-up programs after early obstetrical discharge: an examination of breastfeeding rates, maternal confidence and utilization and costs of health services. *Canadian Journal of Public Health, 94*(2), 98–103. https://doi.org/10.1007/bf03404580	Canada	To determine if there is any difference in outcomes following early obstetric discharge between screening phone calls to identify mothers needing further care and routine home visiting care.	Quantitative - Randomised Controlled Trial.	Screening telephone call to primipara new mother on the first working day post discharge from maternity hospital, to assess her concerns regarding the infant in the areas of feeding, general health, and her emotional health in comparison to routine home visit care.	Telephone call.	*N* = 733 primipara women from two tertiary care hospitals.	In-home postnatal care.	Public Health Nurses.	Effectiveness -No significant differences were found between the two groups in relation to BF and mother's confidence in the first two weeks post-partum. -No significant difference in the infant's medical issues in the first four weeks.
Parker, J., Robinson, J., Mugica-Cox, B., Foy, A., Kepu, K., & Harris-Roxas, B. (2022). How COVID-19 shaped new models of care for a child and family health nursing service. *Australian Journal of Child & Family Health Nursing, 19*(1), 6–14. https://doi.org/10.33235/ajcfhn.19.1.6–14	Australia	To identify any differences in service uptake and the client's perceptions, satisfaction, and perceived benefits of telehealth, and to identify the clinician's experience of telehealth implementation during 6-month pre-COVID period and a 6-month COVID lockdown period.	Mixed methods - (Number of consultations for both 6-month periods and survey feedback regarding telehealth).	Telehealth model of care.	telephone support and video link consultations and groups.	*N*= 9703 (2020) & 9329 (2019) (Total infant child consultations of families with children under the age of 5 years in the South Eastern Sydney Local Health District). The survey was sent to 553 mothers who were involved in a telehealth consultation from the period April to September 2020; 145 responded (26 %).	Community/ In-home.	Child and Family Health Nurses.	Effectiveness Telehealth (video conferencing and telephone support) was an effective and valuable mode of care that provided timely access to families, during a period traditional face to face care was not an option.
Polaschek, L., & Polaschek, N. (2007). Solution-focused conversations: a new therapeutic strategy in well child health nursing telephone consultations. *J Adv Nurs, 59*(2), 111–119. https://doi.org/10.1111/j.1365–2648.2007.04314.x	New Zealand	To explore the perceptions of well-child nurses’ regarding the outcomes of solution-focused telephone consultations with clients.	Qualitative-action research	Solution-focused consultations via a well-child telephone service.	Telephone.	*N*= 10 experienced telephone nurses with a post-basic qualification in well-child nursing.	Community/home - well child telephone line.	Well-child nurses. (health visitors).	Acceptability Permitted clients to -recognize the reason they called/issue of concern -to identify more effective parenting practices to address their specific issue with their child -increased confidence in their own parenting capabilities.
Raatz, M., Ward, E. C., Marshall, J., & Burns, C. L. (2019). Developing the system architecture for conducting synchronous paediatric feeding assessments via telepractice. *J Telemed Telecare, 25*(9), 552–558. https://doi.org/10.1177/1,357,633 × 19,872,091	Australia	To develop and evaluate the required systems architecture to conduct paediatric feeding assessments via synchronous telepractice in patient homes.	Qualitative - A four-phase iterative process (using principles of human-centred design).	Telehealth feeding assessments were conducted in participant homes by a hospital located experienced paediatric speech pathologist.	Video-link.	*N*= 10 typically developing children (aged 1 month to 6 years) and their mothers (*n* = 8).	In the home / from hospital setting.	Speech Pathologist.	Acceptability The iterative study design developed a telehealth system that was acceptable to clients and enabled typical paediatric feeding assessments to be completed in the home.
Robinson, C., Gund, A., Sjöqvist, B. A., & Bry, K. (2016). Using telemedicine in the care of newborn infants after discharge from a neonatal intensive care unit reduced the need for hospital visits. *Acta Paediatr, 105*(8), 902–909. https://doi.org/10.1111/apa.13407	Sweden	To investigate whether the comfort level of parents would be improved using video calls and a web application and if this would decrease the need for hospital visits post discharge from the neonatal intensive care unit.	Quantitative - Randomised Control Trial.	A video call and a web application where the parents answered questions daily and could write to the nurse in addition to standard care 2–3 home visits per week).	Video-link (Skype).	*N* = 137 families were given both oral and written information about the study during their child's hospitalisation. 89 (65 %) consented - 42 families in the control group and 47 families in the study group.	In-home.	Neonatal nurses.	Acceptability -Telehealth accepted by young computer-proficient parents. Effectiveness -telehealth appointment need to reorganise to meet the family's needs. -Telehealth may lead to a more effective use of nursing resources in the home health care of newborn infants.
Rybińska, A., Best, D. L., Goodman, W. B., Bai, Y., & Dodge, K. A. (2022). Transitioning to virtual interaction during the COVID‐19 pandemic: Impact on the family connects postpartum home visiting program activity. *Infant Mental Health Journal, 43*(1), 159–172. https://doi.org/10.1002/imhj.21953	USA	To analyse the impact of virtual home visiting program performance metrics, families' perceptions of virtual home visiting during the pandemic, and the program's activities with marginalised groups.	Quantitative – secondary data analysis of program activity data.	Virtual home visits via phone call 3 weeks post-delivery with a follow-up phone call to address family needs through education, guidance, and community referrals for 4 weeks.	Telephone.	*N* = 7791 scheduled visits.	Integrated home visits.	Registered Nurses.	Effectiveness - families participating comparable. -completion rates decreased by 10.9 % but remained high at 68.1 % of scheduled visits. -impact of virtual visits unknown. -increased community connections for white and affluent families. -decreased community connections for low-income and coloured families.
Seguranyes, G., Costa, D., Fuentelsaz-Gallego, C., Beneit, J. V., Carabantes, D., Gómez-Moreno, C., Palacio-Tauste, A., Pauli, A., & Abella, M. (2014). Efficacy of a videoconferencing intervention compared with standard postnatal care at primary care health centers in Catalonia. *Midwifery, 30*(6), 764–771. https://doi.org/10.1016/j.midw.2013.08.004	Spain	To evaluate the efficacy of incorporating videoconferencing and telephone contact in postpartum care in comparison with standard care.	Quantitative - Multicentre, randomised parallel controlled clinical trial.	Virtual midwifery consultations via videoconference or telephone hotline post-discharge from the hospital.	Videolink (skype) and telephone.	*N* = 1600 (Each of the 8 sites - 200 women - 100 in the IG and 100 in the CG).	Primary Health Care ‘Attention to Sexual and Reproductive Health’(ASSIR) centres, in eight regions of Catalonia (Spain), from November 2008 - December 2009.	Midwives.	Acceptability −453 virtual visits were made by 276 women. -Those who had not used telehealth reported not needing to (83.4 %). -Telehealth reduced the number of visits to the health centre. -increase the reasons for consultations. -timely as mothers could have immediate consultations without having to leave home.
Vicente, D., Venegas, M., Coker, T. R., & Guerrero, A. D. (2022). Promoting Child Development During the COVID-19 Pandemic: Parental Perceptions of Tele-Home Visits in Early Head Start Programs. *Maternal and Child Health Journal, 26*(12), 2496–2505. https://doi.org/10.1007/s10995–022–03,520–4	USA	To understand parental experiences (challenges and satisfaction) of replacing in-person Early Head Start (EHS) home-based services with tele-home visits during the COVID-19 pandemic.	Qualitative - inductive content analysis.	Virtual EHS home-based program.	Video-link (zoom)..	*N* = 35 (parents/mothers)	Virtual home visits	home visitors	Acceptability -Telehealth viable mode of service delivery. Areas to improve the experience include; -provide parenting materials to create an engaging environment for the child/ren. -supplying activities required. -technological support and training for parents.
Wong, M. S., & Chien, W. T. (2023). A Pilot Randomized Controlled Trial of an Online Educational Program for Primiparous Women to Improve Breastfeeding. *Journal of Human Lactation, 39*(1), 107–118. https://doi.org/10.1177/08903344221125129	Hong Kong	To assess the feasibility and effectiveness of a breastfeeding online educational and support program.	Quantitative - Randomized Controlled Trial.	online breastfeeding educational and support program - using BF self-efficacy framework to promote and sustain exclusive breastfeeding.	Video-link (zoom) or telephone call.	*N* = 40 primipara woman (20 in the intervention group & 20 in the control group). NB: 65 % (13) completed the intervention.	Virtual home visits.	Lactation Consultants.	Effectiveness -online education and support antenatally to postnatal could improve exclusive breastfeeding and breastfeeding self-efficacy. Acceptability -It was highly appreciated and valued by participants. -It is accessible and user friendly.

### 2.6 Data analysis and synthesis of findings

A convergent segregated approach was used to synthesise the quantitative and qualitative data. As per the JBI Manual for Evidence Synthesis (Aromataris et al., 2024), this involved separately analysing the quantitative and qualitative studies. We first analysed the quantitative studies' interventions, outcomes, and conclusions, then, where possible, we converged these findings with analysed qualitative findings to link and organise the data to produce an overall analysis of the effectiveness and acceptability of telehealth (Stern et al., 2020). We have presented both quantitative and qualitative research in a narrative form. Quantitative studies are synthesised in a narrative form and analysed according to their intervention, outcomes, and conclusions to determine the effectiveness of telehealth as an intervention. All qualitative studies (including qualitative data from mixed methods papers) were examined for patterns, inductively coded and thematically analysed (Clarke and Braun, 2017) to answer the review research questions. The data analysis process involved familiarisation of the content of the data, manually colour coding initial sets of data, systematically identifying codes preserving the original language used in the papers, developing themes from collated codes and reviewing and defining the thematic analysis (Braun and Clarke, 2006). The socioecological model was used to provide structure for sorting, organising, and reporting findings (Clarke and Braun, 2017). Over several iterations, identified themes were discussed and reviewed by the research team to enhance credibility and trustworthiness (Nowell et al., 2017).

## 3 Results

### 3.1 Descriptive summary

Twenty articles met the eligibility criteria (see Table 1). Study designs included quantitative methods (*n* = 10) mixed methods (*n* = 3) and qualitative (*n* = 7). On quality appraisal, all eligible studies were assessed to be of medium to high quality (Joanna Briggs Institute, 2022), with some limitations discussed below. Most studies (*n* = 7) were from the United States of America (USA) (Agazzi et al., 2021; Beaulieu and Humphreys, 2008; Bock et al., 2021; Ciccia et al., 2011; Guiberson et al., 2015; Rybińska et al., 2022; Vicente et al., 2022), four were from Australia (Gallegos et al., 2018; Morony et al., 2017; Parker et al., 2022; Raatz et al., 2019), three from Sweden (Gund et al., 2013; Lindberg et al., 2009; Robinson et al., 2016) and two from Japan (Furukawa et al., 2020; Kobayashi and Sado, 2019). Single studies were identified from Hong Kong (Wong and Chien, 2023), Spain (Seguranyes et al., 2014), New Zealand (Polaschek and Polaschek, 2007), and Canada (O'Connor et al., 2003). The papers were all published within the last decade, ranging from years 2003–2023.

### 3.2 Acceptability of telehealth

#### 3.2.1 Parental acceptability

The acceptability of telehealth was reported in 13 of the 20 studies included in this review. Four of the five RCTs measured outcomes on parental satisfaction (Gund et al., 2013; Robinson et al., 2016; Seguranyes et al., 2014; Wong and Chien, 2023), when testing interventions comparing standard in-person home care with standard home care and telehealth support. All concluded parental satisfaction with telehealth support in addition to standard care.

Parent satisfaction was also measured in two quasi-RCTs (Beaulieu and Humphreys, 2008; Rybińska et al., 2022). Using secondary program activity data, Rybińska et al. (2022) compared in-person home visits and virtual home visits during the COVID-19 pandemic. They found that families were satisfied with staff communication and reported decreased feelings of needing to seek additional in-person follow-up care after the virtual visit and decreased feelings of isolation during the COVID-19 pandemic. A nurse telephone advice program in a paediatric community clinic found high parental satisfaction with the service (Beaulieu and Humphreys, 2008). The program also found a significant difference in the reasons parents called the telephone advice line, with parents more inclined to seek health care advice post-intervention (Beaulieu and Humphreys, 2008).

Parker et al. (2022), Lindberg et al. (2009), Ciccia et al. (2011), Agazzi et al. (2021) and Vicente et al. (2022) all found telehealth mostly acceptable to parents. Similar to other studies in this review (Gund et al., 2013; Robinson et al., 2016; Seguranyes et al., 2014), Lindberg et al. (2009) found parental satisfaction related to decreased travel, and associated costs and time spent accessing the service. However, videoconferencing was seen to be complementary rather than a replacement for standard care, with face-to-face delivery preferred by some (Agazzi et al., 2021; Parker et al., 2022).

### 3.3 Practitioner acceptability

Three studies reported on the acceptability of telehealth for practitioners (Gund et al., 2013; Morony et al., 2017; Parker et al., 2022). When comparing standard home care and home care supplemented with a video-link call, Gund et al. (2013) concluded that the nurses were mostly motivated to use telehealth, but some were reluctant and avoided the intervention. Similarly, a survey of seven child and family health nurses by Parker et al. (2022) found that whilst telehealth provided access to care and connections with families were maintained during the pandemic, nurses still preferred in-person standard care. Whereas, in assessing a communication technique used on a nursing telephone line, Morony et al. (2017) reported that nurses felt the technique helped to reinforce callers' understanding of the advice given.

### 3.4 Effectiveness of telehealth

The effectiveness of telehealth was reported in 15 of the studies included in this review (see Table 2).

**Table 2:** Outcomes and Effectiveness Findings.

	Outcome	# papers	Citation	Effectiveness Finding
Families	Parental confidence	10	Gund et al., 2013; O'Connor et al., 2003; Robinson et al., 2016; Wong and Chien, 2023; Beaulieu and Humphreys, 2008; Rybińska et al., 2022; Lindberg et al., 2009; Morony et al., 2017; Polaschek and Polaschek, 2007; Gallegos et al., 2018.	Increased Parental Confidence
	Parental satisfaction	4	Gund et al., 2013; Robinson et al., 2016; Seguranyes et al., 2014; Wong and Chien, 2023.	Parent Satisfaction with telehealth
	Breastfeeding rates	4	O'Connor et al., 2003; Seguranyes et al., 2014; Wong and Chien, 2023; Raatz et al., 2019.	Breastfeeding rates between intervention and control groups comparable
	Access to services	4	Robinson et al., 2016; Seguranyes et al., 2014; Kobayashi and Sado, 2019. Parker et al., 2022.	Attendance rates/increased timely access to services
	Parental stress	2	Agazzi et al., 2021; Kobayashi and Sado, 2019.	Reduced parental Stress/better mental health
	Bonding	1	Furukawa et al., 2020.	Increased Father Bonding
	Use of time/money	1	Lindberg et al., 2009.	Increased savings in time/money
	Telehealth use	1	Gund et al., 2013.	Easy to use technology increases the usage of telehealth
Child Health & Development	Language & hearing screening	2	Guiberson et al., 2015; Ciccia et al., 2011.	Language & hearing screening test accuracy
	Preterm discharge health	1	O'Connor et al., 2003.	Health issues post-discharge comparable
	Toddler behavior	1	Agazzi et al., 2021.	Behavior post program comparable
Resources	Cost of telehealth	1	O'Connor et al., 2003.	Decreased cost of telehealth model
	Practitioner workload	1	Kobayashi and Sado, 2019.	Reduced practitioner workload
	Practitioner travel time	1	Bock et al., 2021	Reduced practitioner travel time

In the RCTs (Gund et al., 2013; O'Connor et al., 2003; Robinson et al., 2016; Seguranyes et al., 2014; Wong and Chien, 2023); four measured outcomes on parental satisfaction (Gund et al., 2013; Robinson et al., 2016; Seguranyes et al., 2014; Wong and Chien, 2023); four on parental confidence (Gund et al., 2013; O'Connor et al., 2003; Robinson et al., 2016; Wong and Chien, 2023); and three studies explored breastfeeding outcomes (O'Connor et al., 2003; Seguranyes et al., 2014; Wong and Chien, 2023). Although measuring different outcomes, none of the RCTs found significant differences between the control and telehealth intervention groups, concluding that telehealth may achieve the same outcome as standard care. Noting, that the limitations of the RCTs include the use of self-reported measures in some of the trials, which may have led to social desirability and/or response bias (Wong and Chien, 2023; Seguranyes et al., 2014), low response rates may have introduced selection bias, and retrospective questionnaires may resulted in recall bias (Wong and Chien, 2023; Robinson et al., 2016). Furthermore, O'Connor et al. (2003) experienced issues with generalising results due to different protocols and sample compositions at intervention sites.

For all other studies assessing effectiveness see Table 2.

### 3.5 Implementation enablers and barriers

Four studies discussed enablers and barriers to the implementation of telehealth (Table 3). Ciccia et al. (2011), Vicente et al. (2022), Parker et al. (2022) and Lindberg et al. (2009) all discussed implementation enablers for the families, whilst no studies mentioned enablers for the practitioners. Four studies discussed implementation barriers for families (Bock et al., 2021; Parker et al., 2022; Vicente et al., 2022; Raatz et al., 2019). Two studies address implementation barriers for practitioners (Gallegos et al., 2018; Vicente et al., 2022).

**Table 3:** Telehealth implementation enablers and barriers.

	Enabler	Barrier
Families	No need for travel (Ciccia et al., 2011). Positive relationship with the practitioner (Vicente et al., 2022). Quality of sound and picture of videoconference (Ciccia et al., 2011; Parker et al., 2022). Confidence in using the technology (Lindberg et al., 2009).	Stresses due to COVID-19 (Bock et al., 2021). Not understanding instructions (Parker et al., 2022). Technical issues (Vicente et al., 2022). Flexibility of equipment in a variety of settings (Raatz et al., 2019).
Practitioner		Judgemental language used (Gallegos et al., 2018). Maintaining the child's attention during consultation (Vicente et al., 2022).

### 3.6 Safety of telehealth

Our review did not identify any reports addressing the appropriate use of telehealth or the circumstances under which it may be unsuitable. Furthermore, there were no discussions about clinical governance and safety in the well-child service, nor any studies investigating the potential harms or risks associated with telehealth.

## 4 Discussion

The objective of this systematic review was to explore the evidence base on the use of telehealth in the well-child health setting. Whilst this review found both satisfaction and acceptability of telehealth in targeted interventions for specific programs in the well-child health setting, there is a gap in the literature regarding the effectiveness of telehealth and safety in the universal well-child health setting. No papers discuss when it may be inappropriate to use telehealth, the potential for misdiagnosis when using telehealth or possible adverse events following the use of telehealth.

### 4.1 Telehealth acceptability

Telehealth appears acceptable from the papers examined in this systematic review. This result is consistent with other systematic reviews regarding patient satisfaction with telehealth in the acute health setting (Orlando et al., 2019; Kodjebacheva et al., 2023; Kruse et al., 2017). Orlando et al. (2019) found high levels of consumer satisfaction with telehealth as a mode of service delivery for people living in rural and remote areas, due to the benefits of attending an appointment in a person's community rather than having to travel for the same care in person. This finding was also reported by Kodjebacheva et al. (2023) in a comparison of the satisfaction of telehealth paediatric care and non-telehealth care during the COVID pandemic. Like our review, they concluded that the convenience of telehealth, during the COVID pandemic, resulted in high satisfaction rates for this mode of service delivery (Kodjebacheva et al., 2023). Furthermore, Kruse et al. (2017) reported that telehealth can provide increased access to high-quality services and improve outcomes across a range of medical services, by addressing the barriers of proximity and reducing costs.

As with our review, appropriate technological infrastructure and consumer willingness are also considered key factors for satisfaction with telehealth services in the acute sector (Orlando et al., 2019; Gudipudi et al., 2022). This is supported by Harkey et al. (2020) who report conveniences such as availability of care, reduced costs, safety, and ease of travel as factors influencing client satisfaction with telehealth. Kruse et al. (2017), also conclude that telehealth decreases wait times and missed appointments, therefore improving consumer outcomes. Consumer satisfaction also appears to be related to the interaction between practitioner and consumer (Kodjebacheva et al., 2023). A lack of in-person interaction on telehealth could lead to the consumer thinking the practitioner was less compassionate and the appointments were less personalised. This supports our finding that a positive relationship with the practitioner was an important enabler for telehealth implementation (Vicente et al., 2022).

### 4.2 Telehealth effectiveness

Telehealth may be an acceptable alternative model of care across a range of well-child health interventions however, there is a lack of effectiveness outcome measurement, with most included trials being feasibility, rather than effectiveness RCTs. Furthermore, there are no other systematic reviews that compare the effectiveness of telehealth in the universal well-child health setting regarding developmental assessment, health promotion and prevention for children under six years and age and their families, or the most useful outcomes to assess effectiveness.

In the acute, adult health care setting, there is research that points to telehealth being a satisfactory alternative mode of delivering health care in the acute setting, as it provides choice for patients, can bridge the gap in rural settings and can complement traditional care (Orlando et al., 2019). A systematic review and meta-analyses undertaken by Snoswell et al. (2021), examining primary outcome data, found telehealth to be just as effective as usual care across the disciplines of adult cardiovascular disease, dermatology, endocrinology, neurology, nephrology, obstetrics, ophthalmology, psychiatry and psychology, pulmonary and multidisciplinary care. Snoswell et al. (2021) concluded telehealth to be equivalent or more effective, across the disciplines they included, but more research was needed across different clinical disciplines.

Effectiveness has also been studied in the acute child health setting. A review by Shah and Badawy (2021) of telemedicine in paediatrics, explored evidence of feasibility, accessibility, and satisfaction of telehealth for clients and providers. They also concluded, that whilst more research was required, telehealth was equivalent to or in some cases, more effective than usual care, in the areas of childhood obesity, asthma, mental health, otitis media, skin conditions, diabetes, attention deficit hyperactivity disorder, and cystic fibrosis-related pancreatic insufficiency (Shah and Badawy, 2021).

Whilst our review identified some implementation enablers and barriers, Shah and Badawy (2021) identified several barriers to providers and clients using telehealth, including the need for telehealth training for practitioners. The need for telehealth training is a common finding supported by other disciplines. A study investigating training needs for telehealth for graduate physiotherapists (Martin et al., 2022) found that practical training for telehealth, such as physical assessments, communication, and clinical reasoning, was essential. Gibson et al. (2020) in a study exploring nurse practitioner competence in telehealth, concluded that competency can be increased through exposure in the clinical setting. In a literature review by Fenton et al. (2023), investigating telehealth and mental health training for student nurse practitioners, authors identified components necessary to increase competence and confidence including collaboration and consultation; applicability to practice; and technology use. However, there is a gap in the literature regarding what knowledge skills, and abilities are required to overcome these barriers and effectively implement telehealth in the well-child health setting, with no literature being found.

### 4.3 Safety of telehealth

Our review found no reports on when it may be appropriate or not, to use telehealth. We found no discussions on clinical governance and safety within the well-child service, nor studies exploring the harms of telehealth or the potential risk of harm. Consistent with this finding, a rapid evidence review by Rosen et al. (2023) found no statistical difference in patient safety outcomes in relation to hospitalisation and emergency department visits, when comparing usual care and telehealth. In another review exploring telehealth safety in mental health care, Martiniuk et al. (2023) concluded that telehealth was a safe mode of care, however, more research was needed in understanding adverse events due to telehealth. Similarly, research into increased stillbirths during COVID-19, concluded that there was no reduction in the quality of telehealth antenatal care, but rather potentially a reluctance of women to attend hospital for decreased fetal movements during the pandemic (Hui et al., 2022). Whilst there is a gap in the literature regarding the potential harms of using telehealth, ethical concerns have been raised regarding privacy and confidentiality, due to practitioners being unable to completely ensure privacy and control of the environment during virtual consultations (Keenan et al., 2022).

### 4.4 Implications for clinical practice

Across many acute clinical settings, telehealth is considered an effective healthcare model (Snoswell et al., 2021). However, for children, telehealth research has been limited to treatments of clinical populations with limited research available in well-child health services for infants, young children and their families (Shah and Badawy, 2021). Whilst this review has found that telehealth appears to be an acceptable alternative model of care in the well-child health setting, further exploration is required on the effectiveness, outcome measures, safety and potential harms of telehealth in the well-child health setting to ensure it is a safe and effective model of care.

## 5 Limitations

This study used a comprehensive and systematic approach to review the global literature and used recommended PRISMA reporting guidelines and well established quality assessment tools. However, we restricted our search to English only, high-income countries, and primary research which may have restricted the number of studies identified on the topic. As grey literature on telehealth was not explored, we may have missed papers, introducing publication bias. These methodological issues limit our conclusions, especially regarding the effectiveness of telehealth.

## 6 Conclusion

Early indications suggest that telehealth may be an acceptable mode of service delivery in the well-child health setting, however, there is little evidence of telehealth effectiveness and safety for this setting. Circumstances during the COVID pandemic, highlighted and enhanced the use of telehealth as an alternative to face-to-face services. However, there are significant gaps in the literature regarding the potential harms of telehealth. To be an alternative mode of practice in the well-child health setting, we need to understand when telehealth is effective to use, how to overcome implementation barriers and how to ensure client safety. Therefore, we recommend further research into telehealth safety and, effectiveness to ensure evidence-based policies can be adopted for future well-child health practice.

## CRediT authorship contribution statement

**Kim Howland:** Writing – review & editing, Writing – original draft, Visualization, Methodology, Investigation, Formal analysis, Data curation, Conceptualization. **Kristina Edvardsson:** Writing – review & editing, Validation, Supervision, Resources, Project administration, Formal analysis. **Helen Lees:** Writing – review & editing, Validation. **Leesa Hooker:** Writing – review & editing, Validation, Supervision, Resources, Project administration, Methodology, Formal analysis, Conceptualization.

## Declaration of competing interest

The authors declare that they have no known competing financial interests or personal relationships that could have appeared to influence the work reported in this paper.

## Appendix Supplementary materials

Image, application 1
