# Supplementary material for: Telehealth use in the well-child health setting. A systematic review of acceptability and effectiveness for families and practitioners
Source: Int J Nurs Stud Adv. 2024 Dec 3;8:100277. doi: 10.1016/j.ijnsa.2024.100277 (PMC11681896; doi:10.1016/j.ijnsa.2024.100277)
Supplement: Supplementary file 1 [file mmc1.docx]

**Supplementary File 1 – Search terms**

| **Concept 1:** Well child health Public Nursing setting | **Concept 2:** Telehealth | **Concept 3:** Participants |
| --- | --- | --- |
| Maternal and Child Health Service OR  Maternal and Child Health Nurs* OR  Child and Family health Service OR  Child and Family health nurs* OR  Child Health Service OR  Child Health nurs* OR  Health visitor OR  Karitane nurs* OR  Community health nurs* OR  Public health nurs* OR  Primary Care nurs* OR  Paediatric nurs* | Telehealth OR  Telemedicine OR  Synchronous Telehealth OR  Synchronous Telemedicine OR  Telenurs* OR  Hybrid nurs* OR  Virtual nurs* OR  Online nurs* | Newborn* OR  Infant* OR  Toddler* OR  Preschooler* OR  Child* |
